# Managing laboratory test ordering: a challenge in the new laboratory medicine model

**DOI:** 10.1515/almed-2024-0085

**Published:** 2024-06-25

**Authors:** María Salinas, Ruth Torreblanca, Eduardo Sanchez, Álvaro Blasco, Emilio Flores, Maite López-Garrigós

**Affiliations:** Service of Biochemistry, 16805 Hospital Universitario San Juan de Alicante, Alicante, Spain; Department of Clinical Medicine, Miguel Hernández University, Alicante, Spain; Department of Biochemistry and Molecular Biology, Miguel Hernández University, Elche, Spain; CIBER for Epidemiology and Public Health (CIBERESP), Madrid, Spain

**Keywords:** appropriateness, demand management, result management, laboratory medicine

## Abstract

**Introduction:**

The role of Laboratory Medicine in patient care has evolved in the last decades. The same has occurred to the laboratory model, which has evolved from a traditional model where the laboratory is merely involved in clinical decision-making to a leading model where the laboratory is not only involved but also determines decision-making. The advent of new technologies and automation of processes have enabled laboratory professionals to focus on the first and last phase of the analytical process namely, test ordering and decision-making based on laboratory results. These phases are more error-prone than the analytical phase, and where action must be taken to improve the quality of patient care.

**Content:**

We share our experience in the design and establishment of laboratory test demand management interventions that facilitated diagnosis of occult disease, improved adherence to clinical guidelines, and optimized patient safety.

**Summary:**

A description is provided of key points in the management of laboratory test over/underutilization.

**Outlook:**

The objective of this review is to promote the involvement of laboratory professionals in the design and implementation of demand management interventions and in the development of the new Leader Laboratory model.

## Introduction

Laboratory testing is a multiphase process that starts when the clinician orders a laboratory test and ends when a clinical decision is made on the basis of laboratory results [1]. Laboratory medicine (LM) is the medical specialty most frequently involved [2] in the patient care process. Hence, laboratory reliability is crucial to patient safety [3].

Errors most frequently occur in the pre- and post-analytical phases, as technological advances have significantly reduced errors in the analytical phase [4]. Therefore, measures must be preferentially adopted in the pre- and post-analytical phase to prevent errors and improve patient care [5, 6].

In the last decades, some advances have also contributed to changing the role of LM.

Firstly, the clinical laboratory has become increasingly involved in the patient care process. Forty years ago, the laboratory only was involved in 10–15 % of clinical decisions [7], whereas it is currently involved in the majority of clinical decisions [2].

Secondly, the diagnosis, treatment, prognosis and use or not of particular diagnostic tools are currently established on the basis of specific cut-off values of specific tests. Such is the case of the prostate-specific antigen reference values used to determine whether a biopsy is necessary or not. Thirty years ago, it was unconceivable that the diagnosis of myocardial infarction was established according to the results of a laboratory test, as is the case of troponin [8]. The same occurs to the diagnosis of kidney injury, where diagnosis is based on glomerular filtration rate and urine albumin concentration [9]. Diagnostic laboratory tests must be selected according to evidence-based criteria and clinical guidelines [10].

The last factor that has driven a change in the role of LM is the advent of digital technologies, clinical-decision support (CDS) systems and artificial intelligence [11]. These systems are easily integrated into daily laboratory practice, as laboratories are familiar with the use of information technologies. Although the use of a laboratory information system (LIS) does not necessarily mean that it will be easy to implement and use artificial intelligence-based tools, it makes it easier for laboratory professionals to adapt to these systems.

This change in the positioning of LM also entails a parallel change in the clinical laboratory model and in the role of laboratory professionals. Laboratory professionals have a deeper understanding of laboratory tests than physicians, as Medicine Schools as do not sufficiently teach about them. Despite the advances of the last century, recent studies show that the training of physicians in clinical laboratory sciences still lacks a deep knowledge of biological and analytical variability [12, 13]. The relevance that laboratory results currently have in clinical decision-making requires that laboratory professionals adopt a leading role in the optimal use of laboratory tests and provide knowledge, not only data [14]. It is also laboratory professionals’ responsibility to avoid inappropriate testing and prevent the associated adverse effects [15]. Other competences required of clinical laboratory professionals include creativity, leadership, and communication with the clinician [16]. Communication is essential in any laboratory-led intervention [17]. In addition to the evolution of the role of laboratory professionals, the change in LM positioning also entails a change in the laboratory model [18]. Hence, the laboratory model has transitioned from a traditional or technological model where the laboratory is just an actor involved in clinical decision-making, to a new model, where the clinical decision is determined by the leader laboratory. There are two facts that distinguish a leader laboratory from a traditional laboratory – or from an intermediate laboratory (technological laboratory): (i) the design and implementation of Result Management Interventions (RMIs), by which confirmation is provided that test results have been reported, received and used for adequate action; and (ii) the design and implementation of Demand Management Interventions (DMIs) by which laboratory test over/underutilization is corrected. These two types of interventions do not exist in the traditional model, whereas in the technological model, only DMIs are occasionally implemented to correct overutilization. Differences between these two types of interventions are considerable. For instance, correcting glycated hemoglobin (HbA_1c_) test overutilization only results in a reduction in spending on reagents. In contrast, when testing underutilitzation is corrected by automatically ordering HbA_1c_ testing in all patients – including young patients – with an abnormal lipid profile as recommended by the clinical guidelines [19], this corrective action does not only benefit the patient but society as a whole, as it leads to early diagnosis of diabetes [20]. The Leader Laboratory model facilitates the diagnosis of occult disease, with prognosis improving considerably after early diagnosis.

Additionally, performance indicators are used in this model for monitoring improvements in patient care by the action of the laboratory [21]. In the Leader Laboratory model, other performance indicators are considered in addition to traditional indicators, which could be considered intermediate indicators. Traditional indicators include total number of tests, number of tests per patient, sample-related events, and turnaround time, to name a few. Additional performance indicators considered in the new model include the number of cases of occult disease identified or the increase in the prescription of intramuscular vitamin B12 in primary care (PC) resulting from the identification of cases of severe vitamin B12 deficiency. Therefore, the mission of LM is not only the processing of tests, but the prevention, diagnosis, monitoring and management of disease. This activity is measured by the use of performance – or final – indicators. Hence, laboratory professionals use knowledge, communication, leadership and creativity to fulfill the ultimate mission of a Leader Laboratory: to achieve the maximum benefit for the citizen, the patient and society.

This review is not intended to be an academic treatise on laboratory demand management, nor an exhaustive review of all interventions reported in the literature. The objective of this review is to encourage laboratory professionals to get involved in the design and implementation of DMIs. For such purpose, a description is provided of key points in the design and implementation of DMIs. Then, our experienced authors list and explain the different types of DMIs for correcting laboratory test over/underutilization. All the DMIs implemented have been thoroughly detailed in high-impact journals and are easily replicable. A secondary objective of this review is to encourage laboratory professionals to involve in the development of this new Leader Laboratory model. This model is not only beneficial for citizens, patients and society, but also for the survival of the clinical laboratory itself, which plays a crucial role [22] in the multi-stage patient care process.

## Adverse effects and need to correct inappropriate laboratory test ordering

In an excellent review on the appropriateness of laboratory test requesting, Fryer et al. [23] categorized, defined, and quantified inappropriate request, as follows:

“A request (implying what is ordered by the requestor) that is made outside some form of agreed guidance (including those requested too late).” An inappropriate test request is one that should not be processed, generally because it is requested in the wrong patient, at the wrong time, in the wrong way, or is for the wrong test [24].

Correcting inappropriate requesting is a priority for laboratory professionals, due to its known numerous dramatic adverse effects.

Inappropriate requesting prevents laboratory professionals from fulfilling their mission aimed at the prevention, diagnosis, monitoring and/or treatment of disease. Indeed, failure to order a laboratory test consistent with the clinical status of a patient may lead to missed diagnosis or cause a chronic disease not to be monitored using the laboratory tests recommended in clinical guidelines.

Additionally, test overutilization generates adverse effects beyond costs. Laboratory tests belong to the so-called “little ticket tests” group [25], which are defined as individually “inexpensive” tests that aggregately entail a high cost due to their high demand. Another adverse effect of laboratory test overutilization, apart from increased spending, is the increase in false positives resulting from ordering tests in populations with a low prevalence of the disease [15]. The collateral effects of the Ulysses syndrome [26] and the imaginary invalid syndrome [27] include unnecessary consultations and additional diagnostic tests, generating higher costs than the excessively requested test. The third adverse effect of excessive test ordering is that it contributes to laboratory overutilization. The laboratory becomes a data vending machine rather than a provider of knowledge. The laboratory professional has no time to share knowledge, only data [14]. Hence, a test result potentially useful for adequate clinical decision-making may be overshadowed by irrelevant or supplementary data. This involves a higher risk for the clinician to initiate inappropriate action on the basis of laboratory results.

**Figure 1:**
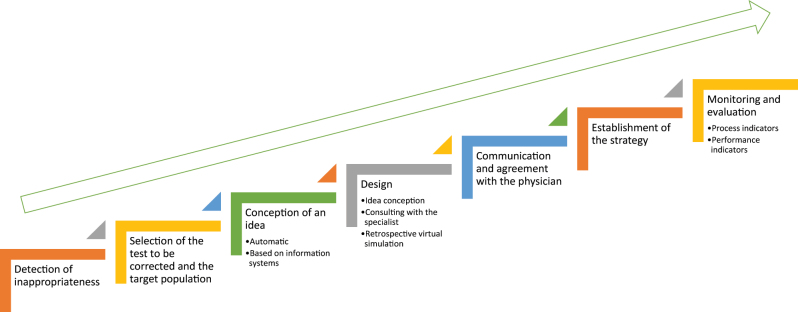
Phases in the design and establishment of demand management interventions.

In the light of the high demand for laboratory tests and the major role of ML in the patient care process, DMIs emerge as crucial for laboratory testing. The design and implementation of DMIs require a deep knowledge and monitoring of all its phases, from the detection of inappropriate test ordering (first phase) to continuous monitoring based on the use of performance indicators (last phase) [28] (Figure 1).

## Phases of the design and establishment of demand management interventions

### Identification of an inappropriate test order

**Figure 2:**
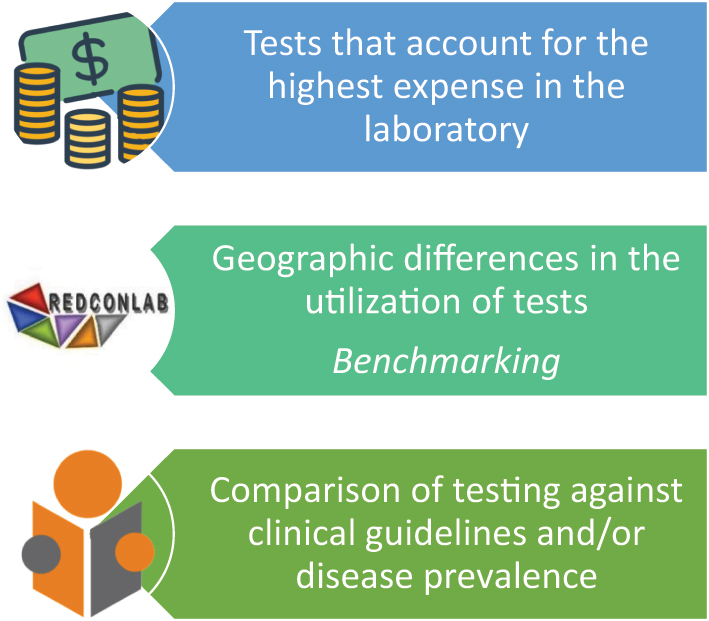
How is inappropriateness detected?

The first phase consists of identifying inappropriate test ordering by evaluating medical records [29] to determine whether or not each request was consistent with the patient’s clinical status. Examination of medical records involves expensive, time-consuming retrospective studies. However, indirect methods have been developed in the recent years for the identification of inappropriate test ordering (Figure 2).

In public health systems, an indirect method is identifying the laboratory tests with a higher impact on laboratory spending. Although a high spending does not mean excessive ordering, it serves as a starting point to determine whether high demand is due to inappropriate test ordering.

Another indirect method to detect inappropriate laboratory test use is to perform comparative studies to identify differences in the use of laboratory tests across different geographic regions. These studies can also help evaluate the consistency of test requests with clinical guidelines, considering the prevalence of the different diseases in our country. These types of studies are carried out by REDCONLAB, a laboratory network of shared knowledge that compares laboratory test requests from PC and hospital emergency departments (EDs).

In the 1st edition of REDCONLAB studies, where only eight laboratories of the Valencian Community took part [30], it was revealed that serum calcium test requests in our health department were far below the volume of requests of other participating health departments. Apart from serum calcium test underutilization, in the same editorial, the journal reported an excessive demand for serum uric acid testing from PC in the eight participating laboratories [31]. This conclusion was drawn on the basis that serum uric acid testing is indicated in case of suspected gout in symptomatic patients. In the light that the prevalence of gout is similar in the two countries, the demand seemed to be very elevated in Spain, as compared to Sweden. The journal also reported a potential excessive demand for iron tests in the eight participating laboratories, since demand for this test was higher than for serum transferrin, which means that serum iron test alone was requested.

In the 3rd and 4th edition of REDCONLAB, where 76 and 110 laboratories participated, respectively, with the 4th edition covering 60 % of the Spanish population, the demand for HbA_1c_ [32, 33] and urine albumin tests [34] was found to be inappropriate. Thus the demand was insufficient for the diagnosis of diabetes and kidney injury, respectively, and the monitoring of patients with hypertension and diabetes, according to clinical guidelines and prevalence of the two diseases in Spain.

In contrast, demand for testing anemia markers, including ferritin, transferrin, vitamin B12 and folic acid [35] and thyroid function markers [36] was excessive.

The 10 tests that generate a higher spending [21] in most of Spanish laboratories coincide with the tests found by REDCONLAB to be excessively requested, including calcidiol [37], ferritin [35], thyrotropin [36], C-reactive protein [38], procalcitonin [39] and liver function markers [40, 41].

The results of these benchmarking studies led to the optimization of laboratory utilization, which demonstrates the usefulness of these studies in the identification and correction of inappropriate test ordering [42]. The experience of the authors indicates that awareness of inappropriateness stimulates the implementation of DMIs. For example, serum calcium test was found by our health department to be underutilized, which drove to the implementation of a DMI to improve diagnosis of primary hyperparathyroidism [43]. This DMI is discussed later in this review.

### Selecting the tests and environment for establishing a DMI

The second stage of the demand adjustment process involves selecting the test which excessive/insufficient demand needs to be corrected and the environment where a DMI needs to be implanted. This stage is crucial for a DMI to be successful.

**Figure 3:**
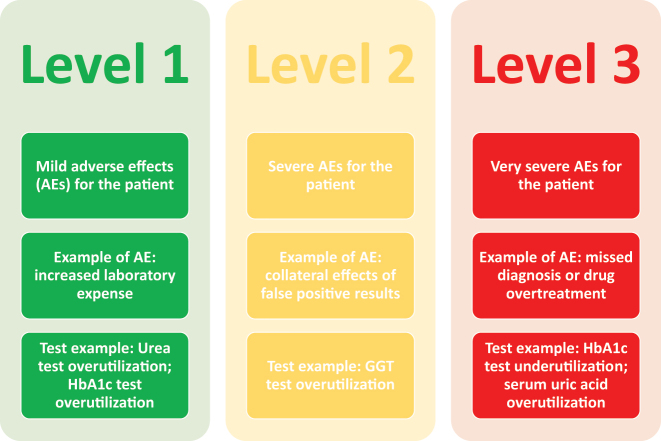
Level of tests according the adverse effects of inappropriate testing. Abbreviations: AE, adverse effects; HbA_1c_, glycosylated hemoglobin.

Inappropriate test orders can be classified as low-risk, intermediate-risk or high-risk, according to the adverse effect they generate (Figure 3). The purpose is to evaluate the risks, consequences or adverse effects of inappropriate test ordering, apart from their economic impact [44]. When failure to order a test prevents early diagnosis, which may potentially cause severe effects (i.e. vitamin B12 deficiency), it is considered high risk. When excessive demand for an inexpensive test only involves a higher spending in reagents without having any relevant impact on patient safety, it is considered low risk.

Once tests have been classified, action is taken to correct demand for high-risk tests [44].

The principle of Pareto is applied to the selection of the environment, otherwise said, the target population of the DMI. Hence, interventions are primarily implemented in PC or EDs as, with a little effort, the design of a single DMI will have a positive impact on many patients.

### Conceiving, pre-designing and designing a DMI

The following consecutive phases are crucial to the demand management process. There are two points in these phases – conception, pre-design and design – that are of paramount importance.

The first point is that DMIs should be automatic and based on information systems. These types of interventions are the only ones whose effects are maintained over time. Educational strategies are initially effective, but they inevitable fall into oblivion over time, even though improvements are shared with clinicians in real time. An example is when C-reactive protein test overutilization was corrected in our hospital. After a consensus protocol was developed by different specialists of our hospital, demand improved to subsequently worsen over time [45].

### The second key point in these phases is communication and consensus with the requesting physician [17]. Establishing a DMI

The next stage involves establishing the DMI. The establishment of a DMI must have a specific duration for the laboratory to be able to analyze its effectiveness and determine whether it should be maintained or not.

### DMI monitoring – process indicators and performance indicators

Last but not least, continuous automatic monitoring of the DMI is necessary. Monitoring is based on process indicators (increase or reduction of demand) and, most importantly, performance indicators. Performance indicators provide information about the improvements achieved with the DMI for the patient, the citizen, and society. These indicators include the number of cases detected during a period of time, the years of life gained in relation to the cases detected, the cost per case detected or the number of unnecessary treatments per case detected. The ultimate goal is to measure the impact of the DMI on the patient, the citizen and society. For instance, the number of cases of acute pancreatitis diagnosed in the ED as a result of the DMI is used as a performance indicator to monitor the effectiveness of a DMI. Another relevant example is the reduction in the number of inappropriate prescriptions of allopurinol when serum uric acid test ordering is optimized.

## DMIs for correcting laboratory test overutilization – adjusting excessive demand

**Figure 4:**
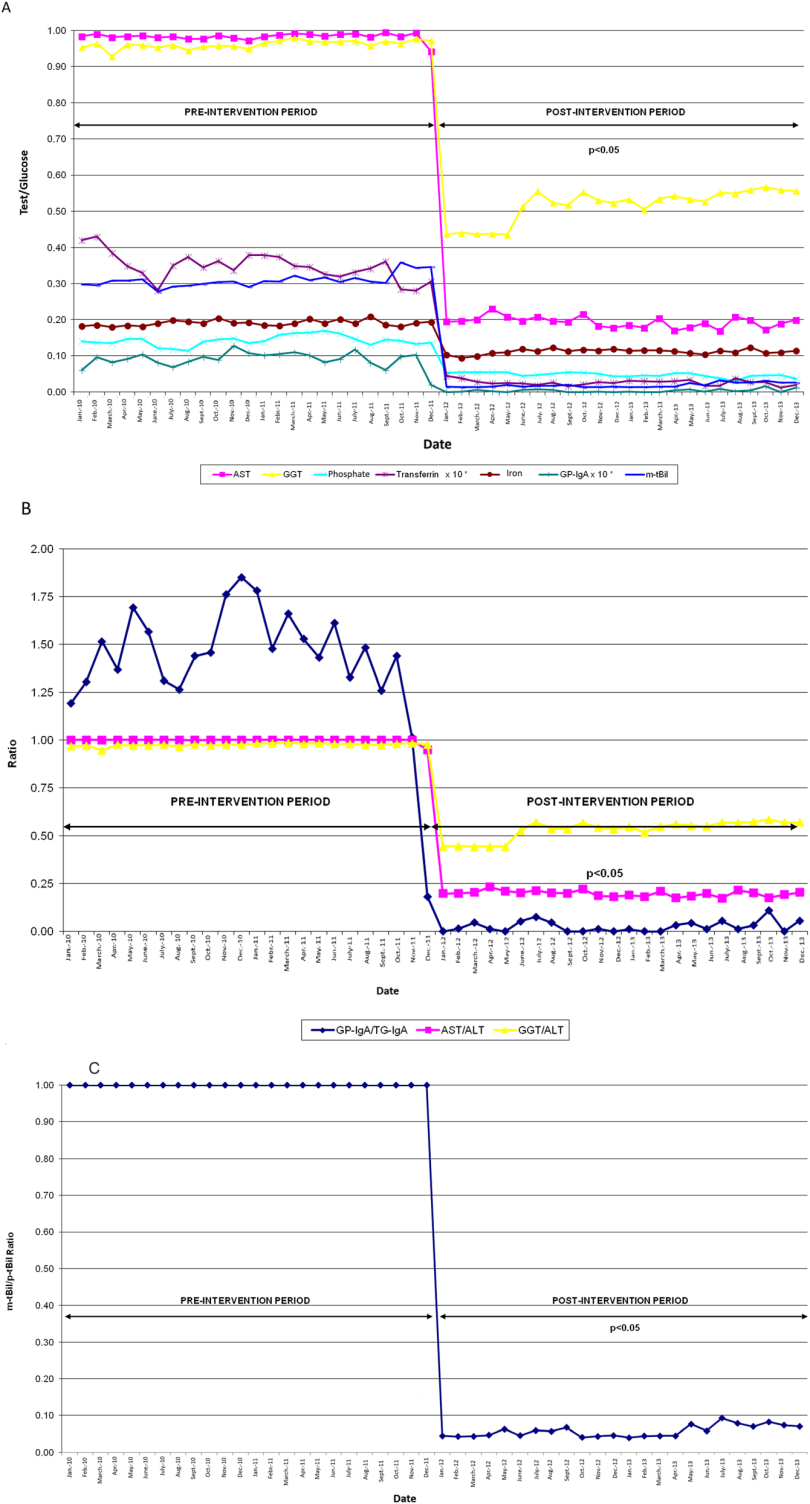
Process indicators. (A) Number of glucose tests. (B) Ratio of related tests. (C) m-tBil/p-tBil. *The value of the transferrin/glucose and GP-IgA/glucose indicator is multiplied by a factor of 10 to avoid the scale effect. AST, aspartate aminotransferase; ALT, alanine aminotransferase; GGT, gamma-glutamyl transferase; GP-IgA, antibodies to deamidated gliadin IgA; m-tBil, measured total bilirubin; p-tBil, total bilirubin ordered; TG-IgA, antibodies to IgA-type tissue transglutaminase.

As aforesaid, DMIs must always be automatic, based on information systems and monitored over time through process indicators. Some examples of DMIs include the first interventions established in our health department [46], whose design is shown in Table 1. These interventions resulted in a decrease in the number of tests (Figure 4). In this case, the reduction of expenditure in reagents was used as a performance indicator.

**Table 1:** Main DMI for correcting excess test requesting.

	Test removed	Profile	Action
**1st group of strategies**			

Removing tests from profiles	AST	Basic clinical profile (ALT, AST, complete blood count, cholesterol, creatinine, GGT, glucose, triglycerides)	AST is added again when ALT has pathological values
		Liver (ALT, AST, GGT, tBil)	
	GGT	Basic health profile	–
	Phosphate	Rheumatology profile (C-reactive protein, calcium, creatinine, complete blood count, ESR, glucose, phosphate, rheumatoid factor).	Phosphate is added again when calcium has pathological values

	**Test removed**	**Condition for removal**	**Action**

**2nd group of strategies**			

Removing tests from orders	Transferrin	If ordered along with ferritin	Transferrin is added again when ferritin >400 ng/mL
	Iron	If not ordered along with ferritin or transferrin	–
	25-OH vitamin D	If the order is not associated with a related diagnosis	–

	**Test changed**	**Condition for changing test**	**Action**

**3rd group of strategies**			

Changing tests	Ig A antibodies against deamidated gliadin	Patient older than 2 years	IgA antibodies against tissue transglutaminase are added

	**Test not performed**	**Condition for not testing**	**Action**

**4th group of strategies**			

Not performing the test	Total bilirubin	If the icteric index is <34.2 mmol/L (2 mg/dL)	A comment is added as an automatic result

All tests refer to serum determinations, except for ESR, which is tested in full blood. ALT, alanine aminotransferase; AST, aspartate aminotransferase; GGT, gammaglutamyltranspeptidase; tBil, total bilirubin; ESR, erythrocyte sedimentation rate.

DMIs based on minimum retest interval (MRI) are very easy to implement as they only require a LIS containing a patient database. There is extensive literature on MRI-based DMIs, with many experiences being based on the MRIs for laboratory tests published by the UK Royal College of Pathologists [47].

MRI is defined as the minimum time before a test should be repeated based on a test properties and the clinical context in which the test is used. It should be taken into account that some tests should only be performed once in life, such as genetic studies or some antibody tests when the result is informative or positive. A MRI may be established for all patients, or for a specific context, such as PC, hospitalization or a combination of the two. Hence, if a test has been ordered a specific number of days or months before, it is not performed, and the previous result is reported in a comment.

Table 2 contains the MRIs that have been established in our laboratory and details the tests involved, date, context where it was applied, and relevant references.

**Table 2:** Minimum re-test intervals.

Laboratory parameters	Scope	Intervention design	Process indicator	Performance indicator	Type of result management
Prostate-specific antigen (PSA)	HOSP	Testing is canceled if ordered in the previous 3 days [28].	Number of tests not processed	Savings	Previous result is reported and a comment is included
	ALL	Testing is canceled if ordered in the previous 3 days.			
Calcidiol (25 hydroxycholecalciferol)	ALL	Testing is not performed if performed in the previous 6 months and previous result was normal.			
Cyanocobalamin (vitamin B12)	ALL	Testing is not performed if performed in the previous 3 months and previous result was normal [48]			
	HOSP	Testing is not performed if performed in the previous 3 days [28]			
HDL cholesterol	HOSP	Testing is not performed if performed in the previous 7 days [28].			
Rheumatoid factor	ALL	Testing is not performed if performed in the previous year.			
	HOSP	Testing is not performed if performed in the previous 3 days [28]			
Ferritin	ALL	Testing is not performed if performed in the previous 3 months and previous result was normal.			
	HOSP	Testing is not performed if performed in the previous 3 days [28]			
Folate	HOSP	Testing is not performed if performed in the previous 3 days [28]			
Iron	HOSP	Testing is not performed if ordered in the previous 3 days [28]			
Immunoglobulins (IGA, IGG, IGM)	HOSP	Testing is not performed if ordered in the previous 3 days [28]			
Transferrin	HOSP	Testing is not performed if ordered in the previous 3 days [28]			
Triglycerides	HOSP	Testing is not performed if ordered in the previous 3 days [28]			
Antinuclear antibodies (ANA)	ALL	Testing is not performed if ordered in the previous 3 months and previous result was normal.			
Extractable nuclear antigen screening	ALL	Testing is not performed if ordered in the previous 3 months.			
Glycated hemoglobin	HOSP	Testing is not performed if ordered in the previous 7 days [28].			
	PC	Testing is not performed if ordered in the previous 2 months.			

HOSP, hospitalized; PC, primary care.

As shown in Table 2, the MRIs established for hospitalized patients correspond to an interval of days. Depending on the test, MRIs range from 3 to 7 days [30], being of months in the other environments. References are not provided for MRIs established by the authors based on their experience. As explained above, once excessive test demand is identified, it is crucial to choose which ones to correct. In our department, the following excessive test requests were identified as high-risk for the patient: serum uric acid, gamma-glutamyl transpeptidase (GGT), and tumor markers from PC. A DMI was designed to correct test overutilization [49–51].

In the case of uric acid, test overutilization entails the risk for treating patients with asymptomatic hyperuricemia. Indeed, the number of allopurinol prescriptions in PC is used as a performance indicator and decreased significantly after excessive uric acid test ordering was corrected [49]. GGT or tumor markers overutilization entails the risk for generating a high number of false positives, with the associated adverse effects (increase in the number of consultations and unnecessary diagnostic tests, iatrogenia, etc.). Furthermore, after alanine aminotransferase (ALT), alkaline phosphatase – not GGT – is the second-level liver function test used to assess cholestasis. Tumor markers should not be used – much less simultaneously – for screening in PC. Our DMI was established based on this premise.

There are some interventions that, although they do not reduce test overutilization, they result in a cost reduction as some values are reported by other low-cost tests. As these interventions only require the use of a LIS, they are easy to implement. Some examples include reporting bilirubin values based on the icteric index [52], and reporting urine albumin based on semi-quantitative results of the creatinine and albumin urinalysis test strip [53].

In the recent years, the laboratory has focused on PC and ED [54]. In the ED, plasma total protein testing was replaced with albumin. Calcium adjusted for albumin is automatically reported in the presence of hyper- or hypocalcemia. Plasma lipase is used as first marker for diagnosis of pancreatitis and the result determines whether amylase is tested or not [55]. Plasma magnesium is automatically measured in the presence of hypocalcemia, hypokalemia or lipase elevation [56].

Finally, when excess test ordering is identified, ONLY the appropriate DMIs should be established to correct it. NEVER stop performing key tests that are useful for the prevention, diagnosis, monitoring and treatment of the disease. For instance, ferritin and vitamin B12 should always be tested even though hemoglobin is normal. The reason is that ferritin is essential for the diagnosis of iron deficiency, whereas vitamin B12 is key to the diagnosis of vitamin B12 deficiency, with many patients having the two deficiencies without concomitant anemia. It is worth noting that dementia in a patient with vitamin B12 deficiency can be irreversible if not treated within the first 6 months [57].

## DMIs for correcting laboratory test underutilization – correcting insufficient demand

As noted above, the adverse effect of laboratory test underutilization includes non-prevention, missed diagnosis, non-monitoring and inadequate treatment, which means that the laboratory does not fulfill its mission.

In most of the laboratory tests both over and underutilization have been noted. This occurs with vitamin B12, whose overutilization was corrected through a MRI-based DMI [48]. Vitamin B12 test underutilization was corrected through a DMI involving automatic test ordering in PC patients when the mean corpuscular volume (MCV) of RBCs was >100 fL [58, 59], added to a DMI for patients receiving chronic treatment with proton pump inhibitors who have not been tested [58].

Another example of test over/underutilization is ferritin. Overutilization of this test was corrected through a MRI-based DMI [60], whereas underutilization is corrected by automatically ordering the test in PC patients with a diagnosis of alopecia [61] or in patients younger than 14 years, though they do not have anemia [62], and iron deficiency is detected. In these two DMIs for correcting test underutilization, vitamin B12/ferritin test was only performed if it had not been ordered or performed in the previous year. Another example of the coexistence of laboratory test over/underutilization is specific IgE test ordering from PC. IgE tests are often requested when they are unnecessary, but they are also frequently missed, so this test requires a DMI to be established [63].

In the new Leader Laboratory model [18], strategies are not only aimed at correcting test overutilization, but also underutilization.

An example of laboratory test underutilization is serum calcium. In PC patients, when serum calcium has not been measured in the previous 3 years and the patient is older than 45 years, it is automatically ordered and tested. In the ED, when a patient has a previous history of cancer, serum calcium test is registered automatically. These two strategies facilitate diagnosis of primary hyperparathyroidism (PHPT) [43] or malignant hypercalcemia [64], respectively. In the DMI for the diagnosis of malignant hypercalcemia at the ED, data on the medical history are accessed in real time via a CDS. The performance indicator used for monitoring progress with the two DMIs is the number of cases of PHPT and malignant hypercalcemia identified, respectively.

Another DMI used for correcting laboratory test underutilization is the automatic ordering and measurement of HbA_1c_ every 3 years in PC patients older than 45 with glucose >110 mg/dL in the same test order [65]. HbA_1c_ is also determined in 25–46 year-old patients with abnormal levels of triglycerides and/or HDL cholesterol, when HbA_1c_ has not been determined in the previous year or when glucose is >100 mg/dL in the same test order [20]. The indicator used for monitoring the effects of these strategies is the number of cases of diabetes identified.

Vitamin B12 test ordering was also corrected [64]. In this case, the performance indicator used is the number of new cases of severe vitamin B12 deficiency identified.

A DMI was also established for magnesium. At the ED, serum magnesium test is automatically registered in patients with hypocalcemia and/or hypokalemia [53, 66]. In PC, apart from these patients, this test is also performed in patients with diabetes and patients older than 65 years when they have not been tested in the previous year [67]. In these interventions, the performance indicator used is the number of patients with hypomagnesemia identified.

As shown above, the laboratory can establish DMIs to detect occult disease. The detection of anti-parietal cell antibodies in patients with severe vitamin B12 deficiency can be very useful [68], since, when positive, it is only performed once in life.

Another strategy that allows screening for occult disease is the identification of patients with myeloma. In PC patients, the LIS automatically orders serum immunoglobulin tests when total serum proteins exceed 80 g/L for the first time. If values of any of the immunoglobulins are above or below their reference values, the LIS automatically orders a serum protein electrophoresis test (proteinogram) [69].

As it is within the scope of laboratory’s mission, it is crucial to adjust test ordering according to clinical guidelines in order to improve disease monitoring. From the laboratory, and in agreement with the physician, HbA_1c_, lipid profile, and urine albumin test ordering can be adjusted in the diabetic patient by ordering these tests according to the intervals indicated in guidelines [20]. Urine albumin testing in patients with hypertension can also be adjusted according to clinical guidelines [70]. With these two DMIs, adherence to clinical guidelines improves, thereby improving the prognosis of a highly prevalent chronic disease. Their efficacy is proven by the reduction of HbA_1c_ concentrations observed in the diabetic patients of our health department [18].

Although these DMIs automatic test ordering resulted in an increased spending on reagents, these interventions were the ones that led to the greatest savings for the clinical laboratory and the health system. Early diagnosis and appropriate monitoring notably improve disease prognosis. In the light that these interventions can be applied to very prevalent diseases, they can be of great benefit to society.

## Conclusions

In the context of the clinical laboratory, the design and implementation of preferably automatic, LIS-based DMIs is critical. Close collaboration with clinicians is essential for ensuring an efficient flow of information. Continuous monitoring based on the use of process indicators is essential, as they reflect the adjustments made to laboratory test ordering. Hence, process indicators show how test ordering was affected by the measures adopted to correct test over/underutilization.

Additionally, the use of performance indicators provides a picture of how these DMIs contribute to improving patient outcomes. In this innovative Leader Laboratory model, whereby the laboratory is not only involved but leads clinical decision-making, the mission of the clinical laboratory goes beyond testing. The focus of the laboratory is placed on disease prevention, diagnosis, monitoring and treatment, which emerge as essential [71].

In this context, the laboratory professional adopts a leading role in the effective management of these systems, thereby contributing to a successful patient-centered healthcare approach.
